# Klotho negatively regulated aerobic glycolysis in colorectal cancer via ERK/HIF1α axis

**DOI:** 10.1186/s12964-018-0241-2

**Published:** 2018-06-08

**Authors:** Qingguo Li, Yaqi Li, Lei Liang, Jing Li, Dakui Luo, Qi Liu, Sanjun Cai, Xinxiang Li

**Affiliations:** 1 Department of Colorectal Surgery, Fudan University Shanghai Cancer Center, No.270 Dong’an Road, Xuhui District, Shanghai, 200032 China; 2 Department of Oncology, Shanghai Medical College, Fudan University, No.270 Dong’an Road, Xuhui District, Shanghai, 200032 China; 3 Departments of CyberKnife, Huashan Hospital, Fudan University, Shanghai, 200032 China

**Keywords:** Colorectal cancer, Klotho, Aerobic glycolysis

## Abstract

**Background:**

Klotho (KL) was originally characterized as an aging suppressor gene, and has been identified as a tumor suppressor gene in a variety of cancers, including colorectal cancer. Recent years have witnessed the importance of metabolism transformation in cancer cell malignancies maintenance. Aberrant cancer cell metabolism is considered to be the hallmark of cancer. Our previous studies demonstrated that KL played negative roles in colon cancer cell proliferation and metastasis. However, its role in the cancer cell reprogramming has seldom been reported. The aim of this study was to examine the role of KL in aerobic glycolysis in colorectal cancer.

**Methods:**

Combining maximum standardized uptake value (SUVmax), which was obtained preoperatively via a PET/CT scan, with immunohistochemistry staining, we analyzed the correlation between SUVmax and KL expression in colorectal cancer tissues. The impact of KL on glucose metabolism and its mechanisms were further validated in vitro and in vivo.

**Results:**

Patients with lower KL expression exhibited higher ^18^F-FDG uptake (*P* < 0.05), indicating that KL might participate in aerobic glycolysis regulation. In vitro assay by using colon cancer cell lines further supported this observation. By overexpressing KL in HTC116 and SW480 cells, we observed that the glycolysis was inhibited and the mitochondrial respiration increased, indicating that KL was a negative regulator of aerobic glycolysis. To seek for the underlying mechanisms, we tried to dig out the relation between KL and HIF1α signaling pathway, and found that KL negatively regulated HIF1α protein level and transcriptional activity. Western blot analysis showed that KL overexpression negatively regulated ERK pathway, and KL regulated aerobic glycolysis in part through its regulation of ERK/ HIF1α axis.

**Conclusions:**

Taken together, KL is a negative regulator of aerobic glycolysis and KL inhibited glucose metabolism transformation via the ERK/ HIF1α axis.

**Electronic supplementary material:**

The online version of this article (10.1186/s12964-018-0241-2) contains supplementary material, which is available to authorized users.

## Background

Colorectal cancer (CRC) is a common cancer and a leading cause of cancer-related mortality and morbidity worldwide [1]. The incidence of CRC is increasing annually and poses a great threat to the health care system. Despite significant progress in the diagnosis and treatment for CRC, the overall survival rate of patients varies from 90% to less than 5% due to different stages of the disease and inadequate diagnosis, and the mortality remains at a high level [2, 3]. Therefore, there is an urgent need for new effective diagnosis and treatment targets and understanding the biology of CRC.

The klotho (KL) gene is a classical ‘aging suppressor’ gene. The role of KL was first demonstrated in the pathology of chronic kidney diseases [4, 5]. The physiological and pathological function of KL shows that it can be used as a regulator of oxidative stress and senescence [6]. Recent studies in cancer demonstrated the KL could be inactivated by promoter hypermethylation and functions as a tumor suppressor [7–9]. KL was reported to be involved in the progression of a series of human cancers, including small cell lung cancer, breast cancer, hepatocellular carcinoma, ovarian cancer, and regulating tumorigenesis, proliferation, progression and resistance to traditional antitumor therapies [10–15]. The canonical roles of KL in cancer are to inhibit fibrosis and promote metastasis via the TGF, Wnt, IGF-I and the bFGF pathways [16–19]. In solid tumors, these signaling pathways are associated with stroma formation and hypoxic microenvironment shaping [20–24]. As is known, solid tumors possess a pronounced hypoxic tumor microenvironment, and these hypoxic conditions pose a threat to tumor cells due to reduced oxygen supply caused by limited vascular system. To survive under the severely hypoxic conditions, tumor cells must reprogram their metabolism pattern [25]. Otto Warburg discovered for the first time that solid tumor cells the metabolism within a solid tumor is significantly different from that of surrounding normal tissue. Over 50% of the cellular energy is produced by glycolysis with the remainder being generated at the mitochondria in tumor cells. This shift occurs even when there is enough O_2_ present to support mitochondrial respiration. This phenomenon is termed as Warburg effect or aerobic glycolysis [26, 27]. The hypoxia-inducible factor 1 (HIF1) transcription factor is perhaps the most important aspect of how cells respond to hypoxic microenvironment and mediate aerobic glycolysis. The net result of hypoxic HIF1 activation is to shift energy production by increasing glycolysis and decreasing mitochondrial function [28]. HIF1 was initially discovered because of its response to low O_2_ concentrations, but now it becomes apparent that HIF1 can be regulated by other factors including oncogene activation or loss of tumor suppressor [29, 30]. Aerobic glycolysis mediated by HIF1 not only provided proliferating cancer cells with building blocks for macromolecule synthesis and energy required in ATP form, but also created an acidic microenvironment caused by lactate that leads to destruction of extracellular matrix that favors metastasis. HIF1 regulated cancer cell proliferation, metastasis, and angiogenesis, thus it becomes a candidate therapeutic target in many cancers [31, 32].

Our previous study demonstrated that KL was a tumor suppressor in CRC and inhibited cell proliferation and metastasis, the malignancies of which were driven by and in part dependent on aerobic glycolysis [17]. However, no studies reported the connection between KL and aerobic glycolysis. Thus, we try to analyze the involvement of KL in aerobic glycolysis clinically and to uncover the underlying mechanisms of HIF1. These findings provide further insight for the anti-tumorigenic role of KL in CRC and raise the possibility that inducing KL expression and inhibiting aerobic glycolysis may provide novel treatment approaches for human colorectal cancer.

## Methods and materials

### Patients and the whole body ^18^F-FDG PET/CT protocol

For PET/CT and Warburg study, 71 colorectal cancer patients who underwent radical surgery between January 2008 and December 2012 at Fudan University Shanghai Cancer Center (FUSCC) were included. Preoperative 18F-FDG PET/CT examination and histopathology confirmation of the presence of colorectal adenocarcinoma were conducted in all patients.

To investigate the mRNA level correlation between KL and HIF1α, a series of 61 patients who received radical resection of primary CRC were included in the study. The tumor samples were put in RNA later and stored in − 20 °C immediately after resection. The demographic and clinical characteristics of the above two cohorts are summarized in Additional file 1: Table S1.

The patients’ in tissue microarray (TMA) has been described previously [33]. All patient studies in our research were approved by institutional review board of FUSCC.

### The whole body ^18^F-FDG PET/CT protocol

The whole-body FDG PET/CT was performed as previously described [24]. Briefly, ^18^F-FDG was made automatically by cyclotron (Siemens CTI RDS Eclipse ST; Knoxville, TN, USA) using an Explora FDG4 module. Patients had been fasting for more than 6 h. Scanning was started 1 h after intravenous injection of the tracer (7.4 MBq/kg). The images were acquired on a Siemens biograph 16HR PET/CT scanner with a transaxial intrinsic spatial resolution of 4.1 mm. CT scanning was first initiated from the proximal thighs to the head, with 120 kV, 80–250 mA, pitch 3.6, and rotation time 0.5 s. Image interpretation was carried out on a multimodality computer platform (Syngo; Siemens). Quantification of metabolic activity was acquired using the standardized uptake value (SUV) normalized to bodyweight and the maximum SUV (SUVmax) for each lesion was calculated.

### Cell culture

The human colon cancer cell lines HCT116 and SW480 were obtained from ATCC and cultured according to the standard ATCC protocols. In brief, HCT116 and SW480 cells were cultured in Dulbecco’s Modified Eagle’s Medium (DMEM), containing fetal bovine serum (FBS) in a final concentration of 10%.

### RNA isolation and quantitative real-time PCR

Total RNA was isolated by using TRIzol reagent (10,296,010, Invitrogen, USA), and TaKaRa’s PrimeScript RT reagent (RR036A) was employed for reverse transcription to obtain cDNA samples. The expression status of designated genes and β-actin were determined by quantitative real-time PCR using an ABI 7900HT Real-Time PCR system (Applied Biosystems, USA). All reactions were run in triplicate. Primer sequences are listed in Additional file 1: Table S2.

### Protein extraction and western blot analysis

Cells were washed twice with ice-dole PBS and lysed in RIPA buffer (150 mM NaCl, 1% NP-40,,50 mM Tris/HCl, pH 8.0 and 10% glycerol) supplemented with 100 μg/ml phenylmethylsulfonyl fluoride (PMSF) for 10 min. Cell debris was removed by centrifugation at 12,000 rpm for 20 min at 4 °C. Protein concentrations were determined by using Thermo Pierce® BCA Protein Assay Kit. 20 μg total protein lysate was subjected to electrophoresis in denaturing 10% SDS-polyacrylamide gel, and then transferred to a membrane for subsequent blotting with antibodies. KL antibody was obtained from Abcam (ab181373). Flag antibody was purchased from Sigma (F1804). Rabbit monoclonal antibody against ERK1/2 (9101), and phosphor-ERK1/2 (4370) were purchased from Cell Signaling Technology. β-actin (60008–1-lg), HIF1α (20960–1-AP), HK2 (22029–1-AP), Glut1 (21829–1-AP), LDHA (19987–1-AP) antibodies that purchased from Proteintech.

### Lentivirus production and stable cell line selection

The Flag-tagged coding sequences of human KL were cloned into pCDH-CMV-MCS-EF1-Puro plasmid (Systembio, SBI) to construct KL expression plasmid. In order to generate KL overexpression cell line, lentiviral particles were produced by co-transfection of pCDH-CMV-MCS-EF1-KL-Puro expressing constructs with psPAX2 and pMD2.G into HEK-293 T cells in a ratio of 4:3:1. Cell lines were obtained by infection of HCT116 and SW480 cells with lentiviral particles followed by puromycin selection.

### Immunohistochemical staining (IHC)

Immunohistochemical staining of paraffin-embedded tissues with antibodies against KL and HIF1α were performed to detect their expression according to standard procedures described previously [34]. In brief, paraffin-embedded sections were routinely baked overnight at 58 °C, de-paraffinized in xylene, rehydrated through graded ethanol, quenched for endogenous peroxidase activity in 0.3% hydrogen peroxide and processed for antigen retrieval by high pressure cooking in citrate buffer (pH = 6.0). Samples were incubated with primary antibodies overnight at 4 °C and secondary antibody for 1 h at room temperature. Diaminobenzidine (DAB) substrate was used for sample immunostaining. Subsequently, sections were counterstained with hematoxylin (Sigma). Anti-KL antibody (Abcam, ab181373) was used in a dilution of 1:100. HIF1α antibody (Proteintech, 20,960–1-AP) was used in a dilution of 1:50. The immunohistochemically stained tissue sections were scored separately by two pathologists blinded to the clinicopathological parameters. The staining intensity was scored as 0 (negative), 1 (weak), 2 (medium) or 3 (strong). Extent of staining was scored as 0 (< 5%), 1(5–25%), 2 (26–50%), 3 (51–75%) and 4 (> 75%) according to the percentages of the positive staining areas in relation to the whole carcinoma area. Scores for staining intensity and percentage positivity of cells were then multiplied to generate the immunoreactivity score (IRS) for each case. Samples having a final staining score of ≤4 were considered to be low and those with score of > 4 were considered to be high.

### Glycolysis analysis

Glucose Uptake Colorimetric Assay Kits (Biovision) and Lactate Colorimetric Assay Kits (Biovision) were purchased to examine the glycolysis process in colon cancer cells, according to the manufacturer’s protocols**.**

### Oxygen consumption rate (OCR) and extracellular acidification rate (ECAR)

Cellular mitochondrial function and glycolytic capacity were measured using the Seahorse Bioscience XF96 Extracellular Flux Analyzer, according to the manufacturer’s instructions of seahorse XF Cell Mito Stress Test Kit or Glycolysis Stress Test Kit. Cells were plated in XF96 Cell Culture Microplates (Seahorse Bioscience) at an initial cellular density of 4 × 10^4^ cells/well the day before determination. Seahorse buffer consists of DMEM medium, phenol red, 25 mM glucose, 2 mM sodium pyruvate, and 2 mM glutamine. For ECAR measurement, 10 mM glucose, 1 μM oligomycin, and 100 mM 2-deoxy-glucose (2-DG) were automatically added to measure ECAR value. After monitoring baseline respiration, 1 μM oligomycin, 1 μM FCCP, and 1 μM rotenone were automatically injected into XF96 Cell Culture Microplates to measure the OCR. The ECAR and OCR values were calculated after normalization of cell number. All experiments were performed in triplicate.

### Analysis of ATP production

The ENLITEN ATP Assay System (Promega, FF2000) was used according to the manufacturer’s instructions. Cells were harvested by scraping and were re-suspended in PBS. The cell suspension was divided into unequal aliquots. Part of the cell suspension was mixed with 5% trichloroacetic acid (TCA). The remaining cells were used for the cell number calculation. Tris-acetate buffer (pH 7.75) was then added to neutralize the TCA and to dilute the TCA to a final concentration of 0.1%. The diluted sample (40 mL) was added to an equal volume of rL/L reagen. Then, luminescence was measured. The ATP standard was serially diluted to generate a regression curve for calculating ATP concentrations in individual samples. The relative ATP concentration was determined and normalized to that of the control cells, which was designated as 1. All experiments were performed in triplicate. Data is represented as mean ± SD.

### Hypoxia response element (HRE) promoter activity with dual luciferase assay

HEK-293 T Cells were seeded into 96-well culture plates and transfected by using Lipofectamine™ 2000 (Invitrogen). 200 ng of pCDH-CMV-MCS-EF1-KL-Puro expressing vector, HRE-luciferase plasmid (Addgene, 26,731) and the Renilla luciferase expression vector, pRL-TK (Promega), were transfected into cells [35]. Forty-eight hours after transfection, cells were assayed for both firefly and renilla luciferase activities using a dual-luciferase system (Promega), as described according to the manufacturer’s protocol. All experiments were performed in triplicate. Data is represented as mean ± SD.

### Statistical analyses

Statistical analyses were performed by SPSS software (version 17.0, IBM Corp., Armonk, NY, USA) using independent *t* test (for continuous variables) and Pearson’s χ^2^ tests (for categorical variables). Statistical significance was based on two-sided *p* values of < 0.05.

## Results

### KL expression is negatively correlated with^18^F-FDG PET/CT SUVmax value

**Fig. 1:**
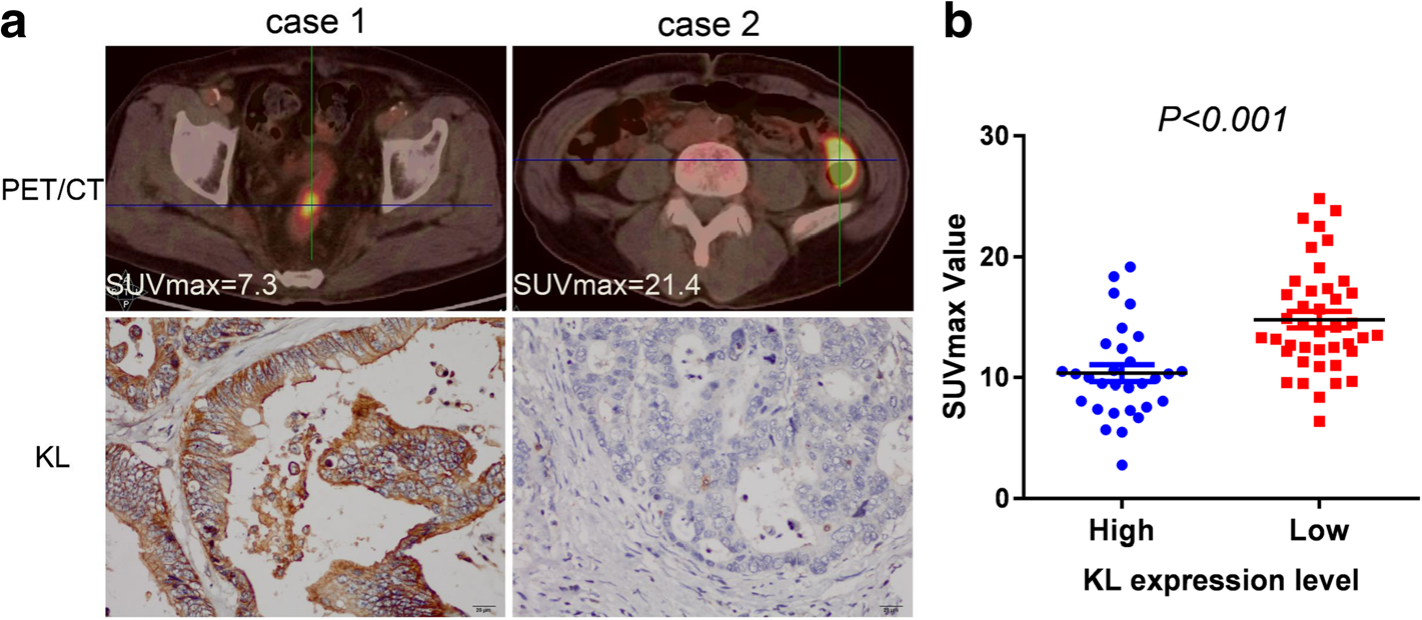
KL expression is negatively correlated with^18^F-FDG PET/CT SUVmax value. In order to assess the contribution of KL expression on metabolic burden, we evaluated the correlation between KL expression and SUVmax value obtained from PET/CT scanning by IHC staining. Representative images from PET/CT scanning in patients with low or high KL expression (magnification scale bar, 20 mm) (**a)**. Analysis of the correlation between SUVmax value with KL expression in KL-Low and KL-High groups of patients (*n* = 71, *p* < 0.001) (**b**)

^18^F-FDG PET/CT, which allows visualization of the metabolic activity of viable tumor cells, has been widely used in the management of cancer diagnosis. SUV_max_ has been widely used as a surrogate marker for the prognosis of numerous types of cancer, including colorectal cancer. In order to explore the clinical relationship between KL and glucose metabolism, we first examine the correlation between KL IHC staining and PET/CT SUV_max_ value. The representative pictures of KL staining were shown in Additional file 2: Figure S1. As expected, colorectal cancer patients with decreased expression of KL exhibited a higher SUV_max_ value (Fig. 1a). Through enlargement of the patients’ sample, we confirmed that the correlation was of statistical significance (Fig. 1b). These results indicate that KL plays a certain negative role in glucose metabolism in colorectal cancer patients.

### KL inhibited aerobic glycolysis in colorectal cancer cells

**Fig. 2:**
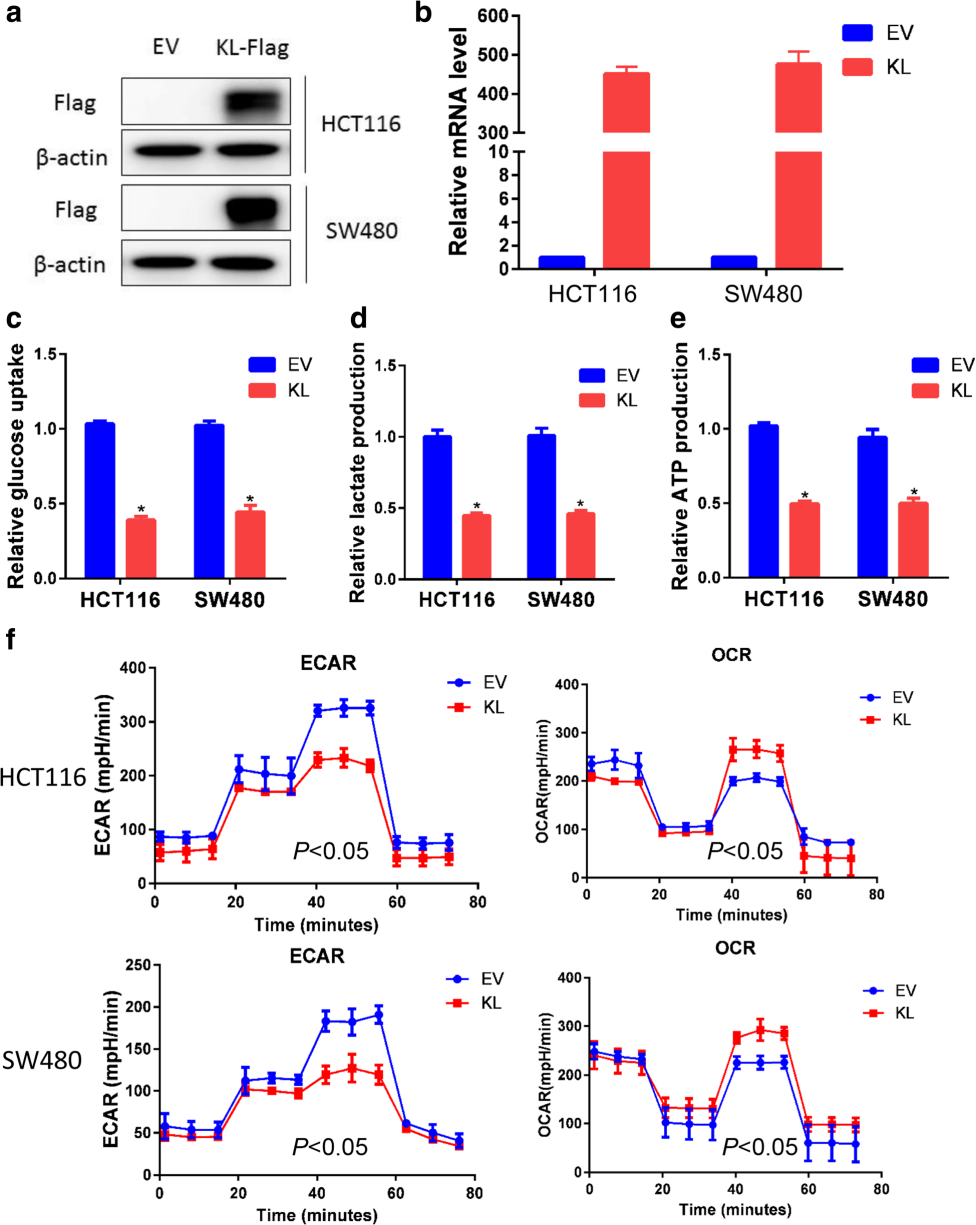
KL inhibited aerobic glycolysis in colon cancer cells. With the aim to assess the role of KL in glycolysis regulation in vitro, as performed a series of in vitro assay. KL overexpressed CRC cancer cells were obtained by using lentiviral mediated transfection, and the efficacy was validated by using immunoblotting with FLAG antibody (**a**) and quantitative PCR (**b**). Forced expression of KL impairs glycolysis in colon cancer cells as determined by reducing glucose consumption (**c**), lactate production (**d**), and ATP production (**e**).Next, the impact of KL on glycolysis rate was assessed by using Seahorse Energy Flux system through examination of ECAR, which reflects the glycolytic rate, and the result suggested that KL overexpression inhibited glycolysis. Mitochondrial respiration, reflected by OCR, usually impaired by glycolysis, and OCR results showed that OCR value increased in KL overexpressed colon cancer cells, further indicating the negative role of KL in glycolysis (f). *P < 0.05

It is generally perceived that proliferated solid cancer cells shifted their glucose metabolism pattern to a hypoxic glycolysis manner. Based on the observation obtained from PET/CT, we suppose that KL may participate in the regulation of aerobic glycolysis. First, we overexpressed KL in HCT116 and SW480 cells, the effect was assayed and confirmed by quantitative PCR and immunoblot (Fig. 2a and b). Second, we examined glucose uptake, lactate production and ATP production, three primary indicators of the Warburg effect. As expected, KL decreased glucose uptake, lactate production and ATP production, indicating its inhibitory role in glycolysis (Fig. 2c-e).

Third, by using Seahorse XF Extracellular Flux Analyzers, we examined the impact of KL overexpression on glycolysis, as reflected by extracellular acidification rate or ECAR. ECAR is an indicator of acidification of the medium surrounding cancer cells that caused by lactic acid, which is a product of aerobic glycolysis. In KL overexpressed HCT116 and SW480 cells, the ECAR decreased significantly, reflecting the negative role of KL in extracellular acidification (*P* < 0.05) (Fig. 2f). Oxygen consumption by cells reflects mitochondrial respiration and could be measured by oxygen consumption rate, namely OCR. In the process of aerobic glycolysis, cells decrease the oxygen consumption rate. In consistence with the ECAR results, we observed a significant increase in the OCR value in KL overexpressed CRC cells (P < 0.05), further reinforced the negative role of KL on aerobic glycolysis (Fig. 2f).

### KL negatively regulated HIF1α protein level and HIF1α transcriptional activity

**Fig. 3:**
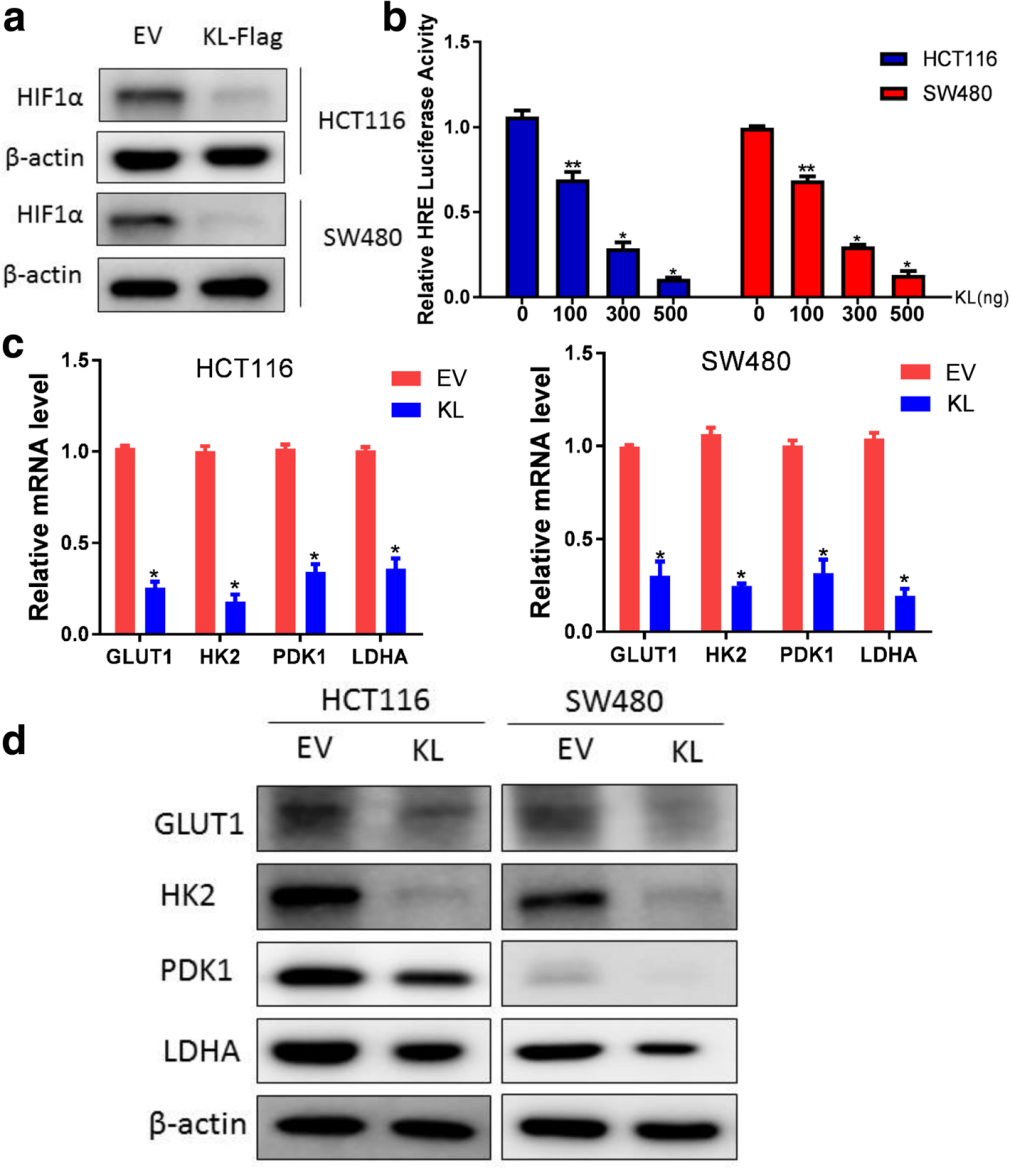
KL negatively regulated HIF1α protein level and HIF1α transcriptional activity. HIF1α is a key factor in regulation of aerobic glycolysis. To answer whether KL regulated aerobic through HIF1α, we first examined the HIF1α protein level in KL overexpressed colon cancer cells and observed a significant decrease in HIF1α protein level (**a**). Then, we performed dual-luciferase assay to assess the contribution of KL on HIF1α transcriptional activity as reflected by HRE-luciferase analysis, and indicated that KL inhibited HIF1α transcriptional activity in a dose-dependent manner (**b**). Glycolysis is a multi-step process involved in the participation of HIF1α targeted glycolytic genes, including GLUT1, HK2, PDK1 and LDHA. In KL overexpressed colon cancer cells, the transcription level of these genes decreased (**c**). Western blot analysis further confirmed the role of KL in down-regulation of the protein level of these genes (**d**). *P < 0.05

HIF1α is a master regulator of aerobic glycolysis and hypoxia adaptation for solid tumors. To assess whether KL regulated aerobic glycolysis via its regulation of HIF1α, we first measured the protein level of HIF1α in KL overexpressed CRC cells. As illustrated, HIF1α protein level decreased significantly when KL expression was introduced (Fig. 3a). Next, we assessed the impact of KL on HIF1α transcriptional activity as reflected by HRE-luciferase activity. We observed that KL negatively regulated HIF1α transcriptional activity in a dose-dependent manner, reflecting a negative role of KL in HIF1α pathway regulation (Fig. 3b). HIF1α regulated aerobic glycolysis via transcription regulation of a series of glycolytic genes, including GLUT1, HK2 and LDHA. Thus, we examined the expression status of these glycolytic genes in KL overexpressed CRC cells. In consistence with the glycolysis analysis, GLUT1, HK2 and LDHA decreased in KL overexpressed CRC cells (Fig. 3c). Western blot analysis further validated the role of KL in regulation of these glycolytic genes (Fig. 3d).

### KL negatively correlated with HIF1α in CRC patients

**Fig. 4:**
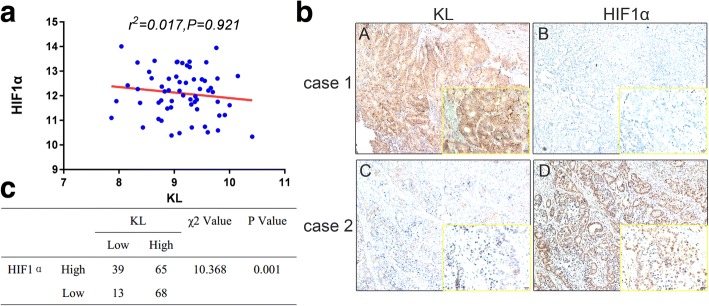
KL negatively correlated with HIF1α in CRC patients. To support the correlation obtained from in vitro assays, we first examined the mRNA of KL and HIF1α, and observed no significant correlation between KL and HIF1α (**a**). Then, we performed IHC staining in CRC patients, and observed a negative correlation between KL and HIF1α in protein level (**b**). Moreover, the correlation was statistically significant, indicating that KL was associated with HIF1α in protein level (**c**)

To support the in vitro observations, we measured the expression status of KL with HIF1α in CRC patients. First, we measured the mRNA level correlation between KL and HIF1α in a series of 61 patients, and observed no significant correlation (Fig. 4a). Then, we examined the correlation between KL and HIF1α in protein levels by IHC staining in TMA including 185 patients. The detailed information of these patients has been described previously (Additional file 3) [33]. As exhibited, HIF1α protein level was higher in patient with decreased KL expression (Fig. 4b). Moreover, the relevance of clinical significance, further supported the negative role of KL in HIF1α signaling pathway regulation (Fig. 4c).

### KL regulates HIF1α in an ERK dependent manner

**Fig. 5:**
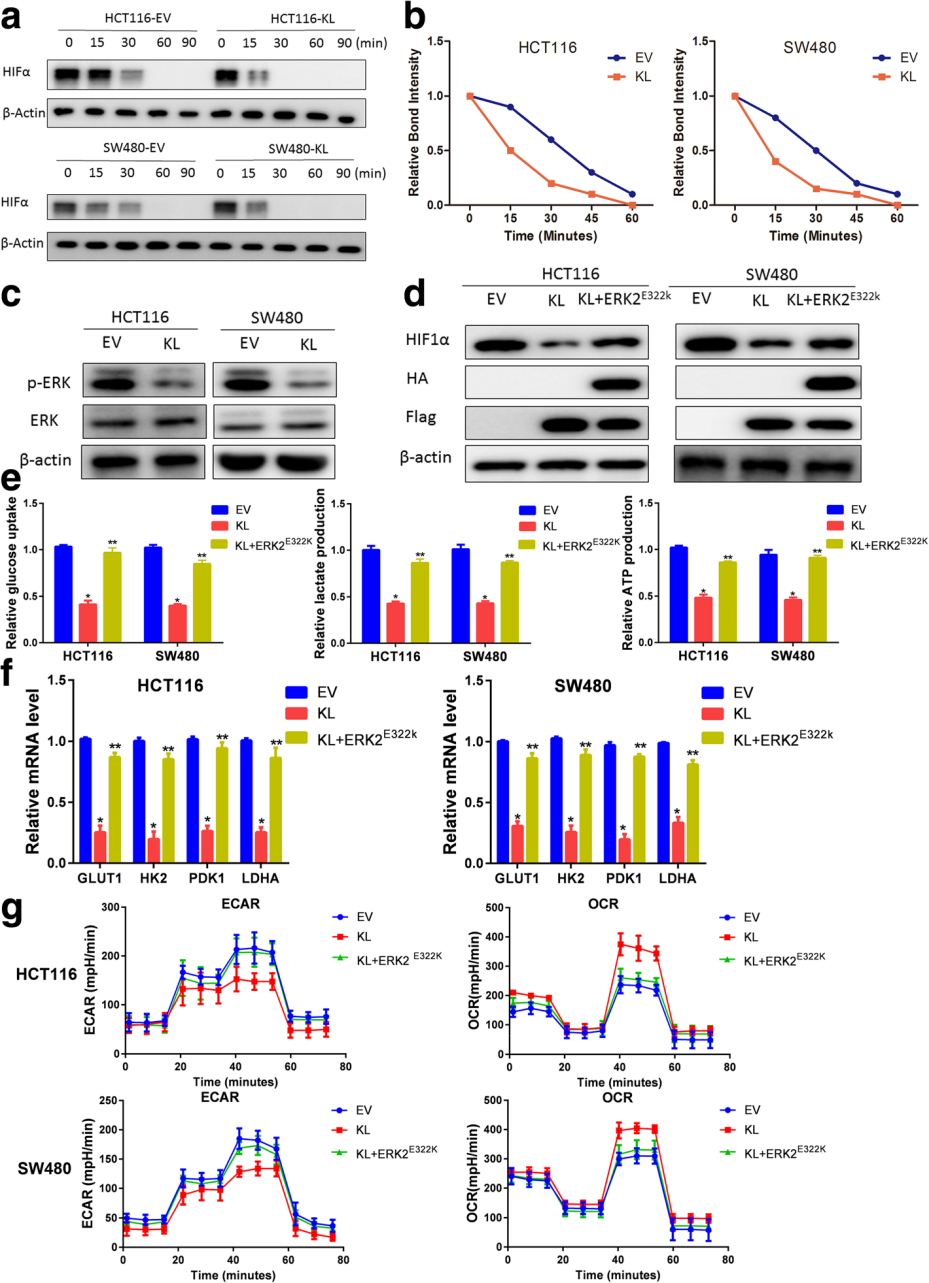
KL regulates HIF1α in an ERK dependent manner. Based on the observation that KL correlated with HIF1α in protein level instead of mRNA level, we assumed that KL might regulate HIF1α stability. First, we examined the hall-life of HIF1α in KL overexpressed HCT-116 and SW480 cells, and results indicated that KL significantly decreased the half-life of HIF1α (**a** and **b**). To seek the underlying molecular mechanism, we examined the activation status of ERK1/2, which regulated HIF1α protein level. In KL overexpressed colon cancer cells, the activation of ERK1/2 was inhibited, indicating that KL might regulate HIF1α via ERK signaling pathway (**c**). To answer whether KL regulated HIF1α via ERK signaling pathway, we overexpressed constitutive activation mutant of ERK2 (ERK2^E322K^) in KL overexpressed cells, and results demonstrated that ERK2^E322K^ introduction could alleviate the decrease in HIF1α caused by KL (**d**). Subsequently, we examined the effect of ERK2^E322K^ on glycolysis in KL overexpressed cells and found ERK2^E322K^ transfection could eliminate the inhibit effect of KL on glycolysis (**e**). Real-time PCR analysis of HIF1α targeted glycolysis genes supported this hypothesis, as ERK2^E322K^ introduction could partially up-regulate the mRNA level of glycolysis genes in KL overexpressed colon cancer cells (**f**). Then, we performed glycolysis analysis, and found that ERK2^E322K^ introduction could induce an increase in ECAR in KL-overexpressed colon cancer cells. In the end, the OCR analysis demonstrated that ERK2^E322K^ introduction could alleviated the attenuation in OCR caused by KL introduction (**g**). Taken together, these results indicated that KL regulated aerobic glycolysis via ERK1/2 activation. *P < 0.05, ** *P* > 0.05

Based on the above observation that KL correlated with HIF1α protein level in CRC patients instead of transcriptional level, we suppose that KL might regulate HIF1α stability. First, we examined the half-life of HIF1α in KL overexpressed CRC cells. As observed, HIF1α half-life was significantly shorter than that in control parent cells, indicating a negative role of KL in HIF1α stability (Fig. 5a and b). Mounting evidence has pointed out that ERK activation was responsible for HIF1α stability maintenance. Then we examined the status of ERK signaling pathway in KL overexpressed CRC cells. We found that KL overexpression inhibited the activation of ERK (Fig. 5c). To determine whether KL regulated HIF1α, we overexpressed constitutive active form of ERK-MAPK kinase ERK2 (ERK2^E322K^) into colon cancer cells. As exhibited, ERK2^E322K^ introduction attenuated the decrease in HIF1α protein level caused by KL overexpression, indicating that ERK pathway activation is responsible for the regulation of HIF1α by KL (Fig. 5d). Subsequently, we examined the effect of ERK2^E322K^ on glycolysis in KL overexpressed cells and found that ERK2^E322K^ transfection could eliminate the inhibit effect of KL on glycolysis (Fig. 5e). Moreover, we examined the expression status of glycolytic genes to assess the impact of ERK on KL/HIF1α axis. As shown, ERK2^E322K^ introduction alleviated the attenuation on the expression of glycolysis genes caused by KL, further supporting our hypothesis that KL regulated HIF1α via ERK activity (Fig. 5f). In the end, we measured the ECAR in ERK2^E322K^/KL overexpression colon cancer cells, and demonstrated that ERK2^E322K^ overexpression increased the ECAR value in KL overexpressed cells (*P* < 0.05), suggesting the role of ERK on KL mediated glycolysis regulation (Fig. 5g). The mitochondrial respiration measurement by OCR test further supported this hypothesis, as ERK2^E322K^ introduction decreased the OCR value that caused by KL overexpression (P < 0.05) (Fig. 5g). Taken together of the present study, we uncovered a novel function of KL in CRC aerobic glycolysis control, and demonstrated that ERK/ HIF1α axis is responsible for KL in aerobic glycolysis regulation. These results provided novel physiological roles for KL and new aspects for the understanding of the biology and treatment of colorectal cancer.

## Discussion

The KL gene was originally identified as a putative aging related suppressor gene in mice, and recovery of KL expression can be used as novel therapeutic strategies for many age-related diseases. KL is expressed most abundantly in the kidneys, but its tissue distribution also includes placenta, prostate, lung, and digestive system [36, 37]. Recent studies demonstrated that due to promoter hypermethylation, the expression level of KL was lower in tumor tissues than adjacent normal samples, indicating that KL may function as a tumor suppressor [7, 38, 39]. In consistence with these observations, the physiological role of KL has been widely studied, and demonstrated that KL played negative roles in oncogenesis, progression and metastasis of many cancers [40–42]. Our previous study also demonstrated that KL functioned as a tumor suppressor in colorectal cancer by inhibiting the IGF1R-mediated PI3K/AKT pathway [17]. Recent years have witnessed the booming of metabolism reprogramming in cancer cells, thus we questioned whether KL could also participate in metabolism control. Besides, KL was reported to regulate ageing relating processes, which also played important roles in metabolism. This also inspired us to question the contribution of KL to metabolism regulation [43–45]. What is more, the IGF1R-mediated PI3K/AKT pathway is also an important cascade in regulating metabolism [46–50]. Based on these hints, we speculated that KL might be associated with metabolism control. Thus, we performed a series of in vitro assays and examination of the correlation between KL with SUVmax reflected by PET/CT scanning, which supported our hypothesis. To search for the underlying molecular mechanism, we turned to examine its effect on HIF1α, a transcription factor that frequently up-regulated in solid tumors.

Solid tumor cells reside in a microenvironment far away from the blood vessels, leading to restricted nutrients and oxygen supply, thus tumor cells evolved an adaptation to survive under hypoxic conditions, known as hypoxic adaptations [51]. The best characterized metabolism reprogramming in cancer is aerobic glycolysis, which is a result of hypoxic adaptations that provided cancer cells with metabolic advantage for proliferation and metastasis. HIF1α played central roles in hypoxic adaptation, and received much attention since its discovery [52]. HIF1α is strictly regulated by a series of post-translational modifications, including acetylation, hydroxylation, phosphorylation and ubiquitination [32, 53]. Previous studies demonstrated that hypoxia activation could induce activation of ERK signaling pathway, and moreover, ERK activity regulated the protein stability of HIF1α [54–56]. Thus, we examined the activation status of ERK1/2 in KL overexpressed colon cancer cells, and observed a decrease in the activation of ERK1/2. Subsequent assays demonstrated that KL regulated HIF1α and glycolysis in an ERK dependent manner. However, there are some issues needed to be addressed in the future, for example, whether the expression of KL changed upon hypoxia, and whether hypoxia lead to epigenetic changes in the promoter region of KL. Furthermore, the impact of ERK activation on KL expression was not examined in the present study, and this also needs to be addressed in the future.

## Conclusions

Taken together, our present study uncovered a novel function of tumor suppressor, KL, in glycolysis regulation and provided the possible molecular mechanism. These results identified KL as a novel target for the diagnosis and treatment for colorectal cancer. Moreover, attempts to target cancer cell metabolism are also novel strategies for the treatment of colorectal cancer, and cutting fuel supply may provide a thoroughly novel aspect in targeting colorectal cancer.

## Additional files

Additional file 1: **Table S1.** Baseline clinicopathological features for patients in PET/CT and RNA study. **Table S2.** Primer sequences used in the study. (DOCX 20 kb)

Additional file 2: **Figure S1.** Representative pictures of each score for immunohistochemical staining results of KL in TMA of FUSCC. (TIF 2829 kb)

Additional file 3: The clinicopathological information and immunohistochemistry results in the TMA cohort of 185 patients. (XLSX 35 kb)

## Abbreviations

CRC: Colorectal cancer
ECAR: extracellular acidification rate
FUSCC: Fudan University Shanghai Cancer Center
HIF1: Hypoxia-inducible factor 1
IHC: Immunohistochemical Staining
IRS: immunoreactivity score
KL: Klotho
OCR: Oxygen consumption rate
SUVmax: maximum standardized uptake value
