# Supplementary material for: Klotho negatively regulated aerobic glycolysis in colorectal cancer via ERK/HIF1α axis
Source: Cell Commun Signal. 2018 Jun 8;16:26. doi: 10.1186/s12964-018-0241-2 (PMC5994118; doi:10.1186/s12964-018-0241-2)
Supplement: Supplementary file 1 — Table S1. Baseline clinicopathological features for patients in PET/CT and RNA study. Table S2. Primer sequences used in the study. (DOCX 20 kb) [file 12964_2018_241_MOESM1_ESM.docx]

**Table S1. Baseline clinicopathological features for patients in PET/CT and RNA study**

| **Variable** | **PET/CT study** | | **RNA study** | |
| --- | --- | --- | --- | --- |
|  | **n** | **%** | **n** | **%** |
| **Gender** |  |  |  |  |
| Male | 48 | 67.61% | 33 | 54.10% |
| Female | 23 | 32.39% | 28 | 45.90% |
| **Age** | 56 (24-86) | | 58(18-86) | |
| **T category** |  |  |  |  |
| T1/T2 | 14 | 19.72% | 17 | 27.87% |
| T3/T4 | 57 | 80.28% | 44 | 72.13% |
| **LN status** |  |  |  |  |
| Negative | 30 | 42.25% | 24 | 39.34% |
| Positive | 41 | 57.75% | 37 | 60.66% |
| [**Pathological grading**](http://dict.cn/pathological%20grading) |  |  |  |  |
| High/ Moderate | 50 | 70.42% | 43 | 70.49% |
| Poor/ [undifferentiation](http://dict.cn/undifferentiation) | 21 | 29.58% | 18 | 29.51% |
| **Lymphovascular invasion** |  |  |  |  |
| Negative | 46 | 64.79% | 47 | 77.05% |
| Positive | 25 | 35.21% | 14 | 22.95% |
| **Perineural invasion** |  |  |  |  |
| Negative | 55 | 77.46% | 48 | 78.69% |
| Positive | 16 | 22.54% | 13 | 21.31% |
| **SUVmax** | 12.92 (2.80-24.80) | | - | |

**Table S2. Primer sequences used in the study.**

| Primer | Sequence |
| --- | --- |
| KL forward | 5’- TGAGGACGACCAGCTGAGGGTGTAT-3’ |
| KL reverse | 5’- CATGGATGCCTTGGGCTCAAACTG-3’ |
| Glut1 forward | 5’- TGTCGTGTCGCTGTTTGTGGTGGA -3’ |
| Glut1 reverse | 5’- TGAAGAACAGAACCAGGAGCACAG -3’ |
| HK2 forward | 5’- TGATGTGGCTGTGGATGAGCT -3’ |
| HK2 reverse | 5’- GCCAGGCAGTCACTCTCAATCTG -3’ |
| PDK1 forward | 5’-GACTGTGAAGATGAGTGACCGAGG-3’ |
| PDK1 reverse | 5’- CTATTGAGTCTGTTGACAGAGCC-3’ |
| LDHA forward | 5’- AGGCTGGGAGTTCACCCATTAAGC -3’ |
| LDHA reverse | 5’- GAGTCCAATAGCCCAGGATGTG -3’ |
| β-actin forward | 5’- CTACGTCGCCCTGGACTTCGAGC-3’ |
| β-actin reverse | 5’- GATGGAGCCGCCGATCCACACGG-3’ |
