# Supplementary figures and images for: Klotho negatively regulated aerobic glycolysis in colorectal cancer via ERK/HIF1α axis
Source: Cell Commun Signal. 2018 Jun 8;16:26. doi: 10.1186/s12964-018-0241-2 (PMC5994118; doi:10.1186/s12964-018-0241-2)

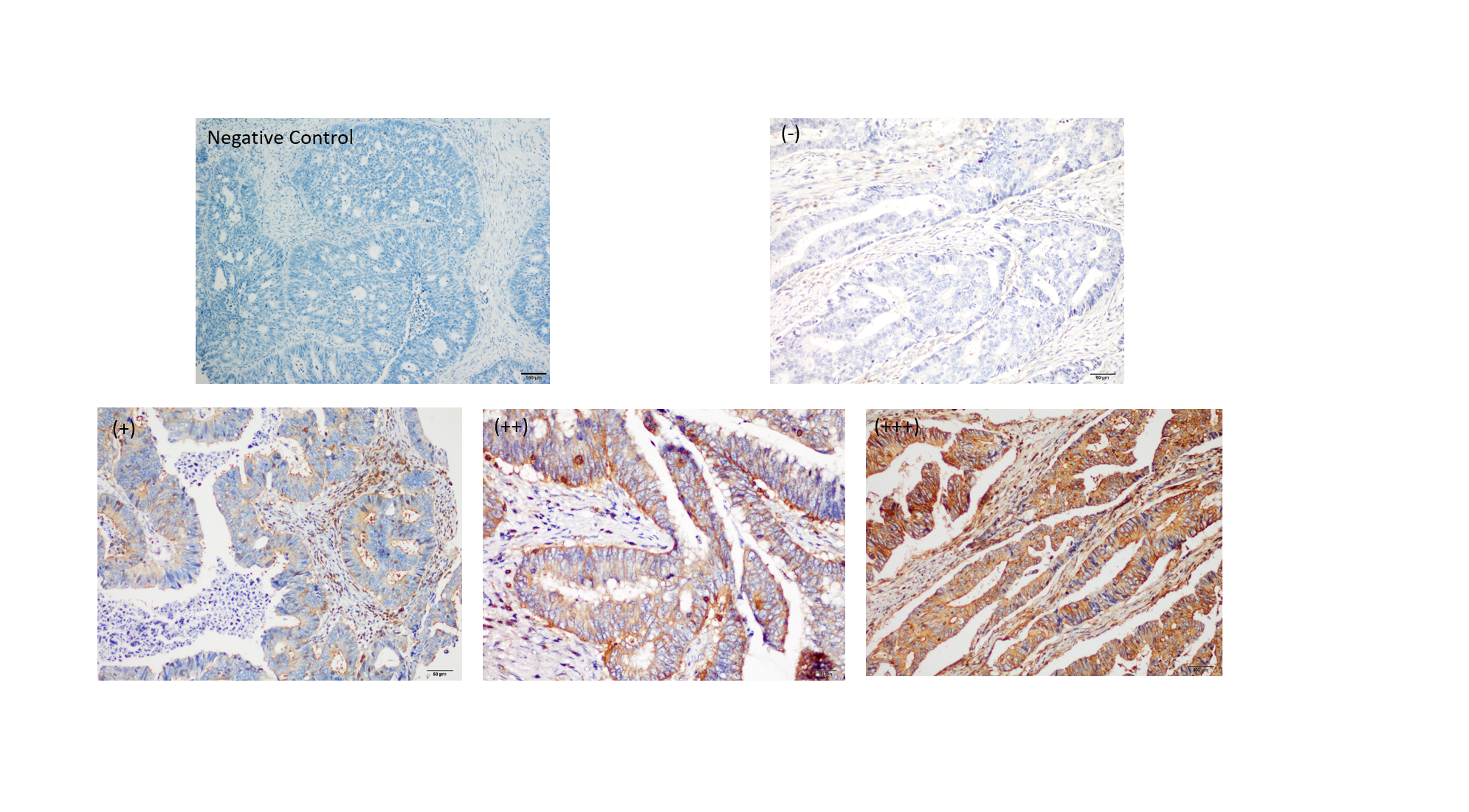

Supplement: Supplementary file 2 — Figure S1. Representative pictures of each score for immunohistochemical staining results of KL in TMA of FUSCC. (TIF 2829 kb) [file 12964_2018_241_MOESM2_ESM.tif]
